# The effects of different types of Tai Chi exercises on preventing falls in older adults: a systematic review and network meta-analysis

**DOI:** 10.1007/s40520-023-02674-7

**Published:** 2024-03-13

**Authors:** Jiaqi Lin, Shuaiqi Ning, Shaowei Lyu, Hainan Gao, Xinxin Shao, Zili Tan, Xiangyu Zhu, Ying Chen

**Affiliations:** 1 School of Acupuncture-Moxibustion and Tuina, Beijing University of Chinese Medicine, Beijing, China; 2 Department of Encephalopathy, Beijing Dongzhimen Hospital, Beijing University of Chinese Medicine, Beijing, China

**Keywords:** Older adults, Exercise therapy, Falls, Network meta-analysis, Tai Chi

## Abstract

**Objectives:**

Few studies comparing the effects of different types of Tai Chi exercises on preventing falls in older adults. We compared the effects for finding an optimal intervention.

**Methods:**

We searched 12 databases, including PubMed, EMBASE, Cochrane Library, Chinese National Knowledge Infrastructure (CNKI) and so on, from their inception to January 13, 2023. Randomized controlled trials incorporating different types of Tai Chi for preventing falls in older adults were included. The outcome measures were the incidence of falls and Berg Balance Scale (BBS). Network meta-analysis (NMA) was conducted using Stata 15.1 based on a frequentist framework.

**Results:**

Seventeen trials were eligible, including 3470 participants and four types of Tai Chi. They were 24-form simplified Tai Chi (24-form), Yang style Tai Chi (Yang style), Sun style Tai Chi (Sun style) and Tai Chi exercise program (TCEP). In paired meta-analysis, for incidence of falls, 24-form (Relative Risk (RR) = 0.59, 95% confidence interval (CI) [0.40, 0.86]) was more efficient than the control group. For BBS outcome, 24-form (MD (mean difference) = 2.32, 95% CI [1.42, 3.22]) was better than the control group. In the NMA, the results of incidence of falls were as follows: 24-form > Yang style > Sun style > control > TCEP. The rank probability of BBS was as follows: 24-form > TCEP > Yang style > control.

**Conclusion:**

Among the four types of Tai Chi studied, the 24-form simplified Tai Chi has shown better efficacy than other types.

## Introduction

The World Health Organization defines older adults as those who are 60 years of age or older. Human aging has the following manifestations in the musculoskeletal system: After the age of 35, the imbalance of calcium metabolism leads to the dissociation of bone tissue more than its formation, which reduces bone density. Bones become brittle and thin, making people prone to fractures [1, 2]. For cartilage, it hardens and loses elasticity, reducing joint flexibility [3]. Muscle fibers atrophy and lose their elasticity [4, 5]. Older adults’ movements start to become slower and they are easier to fall [6].

Falls is defined as “unintentionally coming to rest on the ground, floor, or other lower level [7].” Falls occur at all ages. According to statistics, they occur in 30% of adults aged over 65 years annually [8]. Even though most falls do not result in death, they can cause serious injuries such as fractures and brain damage. Compared to older adults who did not fall, falling older adults had greater declines in activities of daily living, physical health, social activities, spirit and psychology [9].

Therefore, if older adults do not receive interventions to prevent falls, their quality of life will be threatened. Exercise is one of the non-pharmaceutical methods that effectively prevents falling and improve the dynamic balance ability of older adults [10, 11]. Exercise can improve the muscle strength, self-pain perception, physiological function and independent living ability of older adults [12, 13]. Tai Chi, one of traditional Chinese exercise, is a suitable exercise for them [14]. It is characterized by a series of gentle, low-impact and coordinated movements [15]. The rhythm of Tai Chi practice is slow. Regular practice of Tai Chi helps to improve coordination and balance of movements. Long-term Tai Chi practice not only enhances the participants’ lower limb muscular endurance, but also improves neuromuscular control and proprioception of the knee and ankle joints [16, 17]. A study found that long-term Tai Chi practitioners had significantly increased cortical thickness in the prefrontal and premotor cortexes of brain. This suggested that long-term Tai Chi training had a positive effect on the optimization of brain structure and function [18].

Our study included four types of Tai Chi. They were 24-form simplified Tai Chi (24-form), Yang style Tai Chi (Yang style), Sun style Tai Chi (Sun style) and TCEP.

The 24-form simplified Tai Chi, also known as Simplified Tai Chi, was developed by China National Sports Commission through extracting the essence of Yang style Tai Chi in 1956. Although it has only 24 forms, it is more refined and standardized than the traditional Tai Chi routines. The 24-form simplified Tai Chi can fully reflect the movement characteristics of Tai Chi.

Yang style Tai Chi is distinguished by soft and gentle movements and a well-stretched body. The basic movements include Commencing form, Part Wild Horses Mane, Single Whip, Wave Hands like Clouds, Repulse Monkey, Brush Knees, Fair Lady Works at Shuttle, Grasp Peacocks Tail and Closing Form [19]. Compared with 24-form simplified Tai Chi, it has fewer movements and can be learned in a short time.

The movements of Sun style Tai Chi incorporate Yi and Bagua. It moves at a tight pace. When moving one foot forward, the other foot will follow at the same time, and the same goes for the retreat. Turning from side to side is connected with open-close movement. Sun style Tai Chi can gradually improve the adaptability by increasing the level of homeostasis of physical and mental system [20].

Finally, it is regarding TCEP. The trials would be classified as TCEP when older adults participated in Tai Chi exercises which specially formulated by researchers to prevent falling. For example, three studies [11, 21, 22] drew on classical movements from Tai Chi routines and then modified them in combination with participants’ individual abilities. One study [23] only selected single movements of Tai Chi routines.

However, there is no adequate evidence to suggest which type of Tai Chi is the most effective on preventing falls in older adults. Therefore, our study conducted a network meta-analysis (NMA) to investigate the efficacy of different types of Tai Chi on preventing falls in older adults.

## Methods

### Protocol and registration

This systematic review and NMA were strictly conducted according to the Preferred Reporting Items for Systematic Review and Meta-analysis for Network Meta-analysis (PRISMA-NMA) [24]. Moreover, we have registered the protocol of this review in International Prospective Register of Systematic Reviews (PROSPERO) (CRD42022355710) in advance.

### Eligibility criteria

#### Inclusion criteria

The inclusion criteria were as follows: English or Chinese literature; randomized controlled trials (RCTs); The study population was older adults (≥ 60 years) who did not have any musculoskeletal or neurological problems that affected limb function. There were no restrictions on gender, nationality or race; Falls was defined as “unintentionally coming to rest on the ground, floor, or other lower level”; Tai Chi was used to improve balance function or reduce falls; the duration, frequency or style of Tai Chi intervention was not limited.

#### Exclusion criteria

The exclusion criteria was as follows: studies that duplicate published; studies that were not RCTs or protocols; older adults with stroke, Parkinson’s disease, Cognitive impairment, osteoporosis, osteoarthritis, diabetes, dementia, or any other musculoskeletal or neurological problem that affects the limb function; experimental groups used non-Tai Chi therapy; studies language that was not Chinese or English; the full text was not available; the original study data were missing or data could not be extracted.

### Data sources and search strategy

The academic articles were systematically retrieved following the PRISMA-NMA statement. Two reviewers (JQL and SQN) independently searched 12 databases, including PubMed, EMBASE, Cochrane Library, Chinese National Knowledge Infrastructure (CNKI), Wanfang Database, Chinese Scientific Journal Database (VIP) and so on, from their inception to January 13, 2023. The search strategy for PubMed database was presented in Table 1.

**Table 1:** Search strategy in PubMed

Search	Query
#1	(“Aged”[Mesh]) OR ((((((((elderly[Title/Abstract]) OR (older[Title/Abstract])) OR (elder[Title/Abstract])) OR (geriatric[Title/Abstract])) OR (elderly people[Title/Abstract])) OR (old people[Title/Abstract])) OR (senior[Title/Abstract])) OR (aging[Title/Abstract]))
#2	(“Tai Ji”[Mesh]) OR ((((((((((Tai-ji[Title/Abstract]) OR (Tai Chi[Title/Abstract])) OR (Chi, Tai[Title/Abstract])) OR (Tai Ji Quan[Title/Abstract])) OR (Ji Quan, Tai[Title/Abstract])) OR (Quan, Tai Ji[Title/Abstract])) OR (Taiji[Title/Abstract])) OR (Taijiquan[Title/Abstract])) OR (T’ai Chi[Title/Abstract])) OR (Tai Chi Chuan[Title/Abstract]))
#3	(“Accidental Falls”[Mesh]) OR ((((((Falls[Title/Abstract]) OR (Falling[Title/Abstract])) OR (Falls, Accidental[Title/Abstract])) OR (Accidental Fall[Title/Abstract])) OR (Fall, Accidental[Title/Abstract])) OR (fall[Title/Abstract]))
#4	randomized controlled trial[Publication Type] OR randomized[Title/Abstract] OR placebo[Title/Abstract] OR RCT[Title/Abstract] OR Random[Title/Abstract]
#5	#1 AND #2 AND #3 AND #4

### Data extraction and quality assessment

We input the records from electronic databases into database management software, and the duplicate citations were removed. Data extraction was performed by two reviewers (JQL and SQN) who independently evaluated the eligibility of the remaining citations by examining the titles, abstracts and full articles sequentially. The name of the first author, year of publication, sample size, age, type of Tai Chi (TC) treatment, details of the control intervention, duration of TC treatment, and primary outcome were recorded for each RCT. The first author should be contacted to obtain relevant data whenever possible. Two reviewers (JQL and SWL) assessed the quality of included RCTs using the Cochrane risk of bias tool, which focuses on random sequence generation (selection bias), allocation concealment (selection bias), performance bias, detection bias, attrition bias, reporting bias, and other bias. Each bias was judged as low risk, unclear, or high risk in terms of the guidelines. Disagreements were resolved through discussions between the same reviewers. If the issue still persisted, a third reviewer (XYZ) was consulted and then made the final decision.

### Outcome measures

The outcome measures were incidence of falls and Berg Balance Scale (BBS) [25]. Incidence of falls is the most direct indicator of effectiveness, and therefore it is the most commonly used outcome measure. The second commonly used is BBS. The most important factor affecting falls in older adults is balance. BBS can evaluate a person’s balance function through completing 14 tasks with different levels of difficulty. Each task score ranged from 0 to 4, and the total score ranged from 0 to 56, with higher scores indicating better balance.

### Data synthesis and analysis

Data analysis was performed using Stata (Version 15.1; StataCorp., College Station, TX, USA). For the incidence of falls, RR (Relative Risk) values and 95% confidence interval (CI) were used. BBS results were used with mean difference (MD) and 95% CI. Firstly, the paired meta-analysis was performed. To assess the heterogeneity among studies, we calculated *I*^*2*^. Then, the NMA within the frequentist framework was generated using Stata to plot the network diagram and interval forest plot. The inconsistency model test was applied, and if *p* > 0.05, the consistency model was applied for analysis. Clinical significance was considered insignificant, if the invalid vertical line intersected the horizontal line of the 95% CI. The surface under the cumulative ranking curve (SUCRA) was used to reflect the effectiveness rank of the five different treatments. The larger the area under the curve (0–100%), the better the treatment effect. Finally, funnel plots were created to check for publication bias. Egger’s test was performed at a significance level of 0.1. The stability of the results was reliable when the funnel plots for most of the included studies were vertically distributed to the midline (x = 0) [26].

## Results

### Study selection and characteristics

**Fig. 1:**
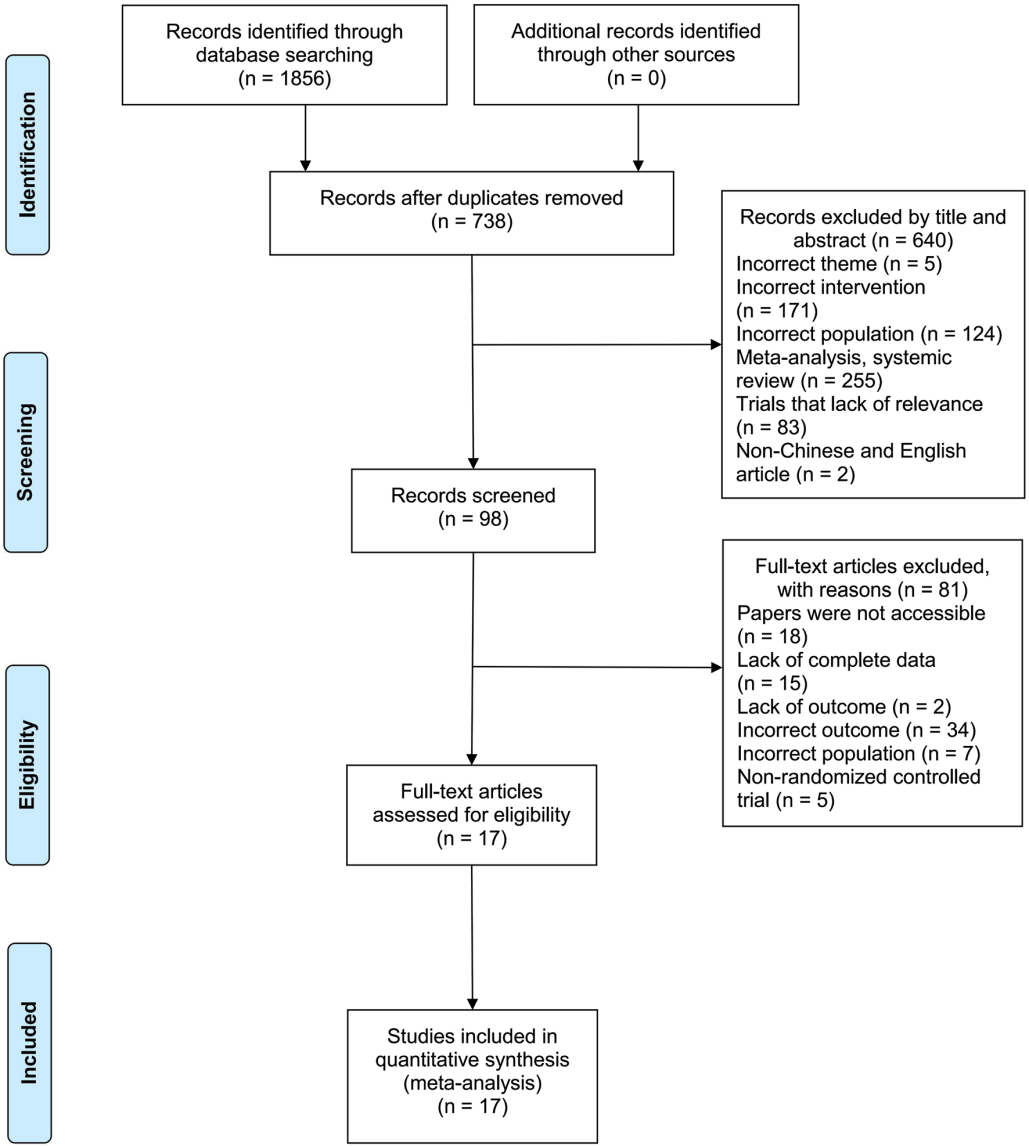
Flowchart of the study selection process

A total of 1856 studies were retrieved from twelve databases. After removing duplicates, 738 articles were included. After reviewing the titles and abstracts, 640 articles were excluded. A full review of the remaining 98 articles excluded 81 irrelevant articles, including 18 inaccessible articles, 15 articles with incomplete outcome data, 2 articles that did not report outcomes, 34 articles that did not report the incidence of falls or BBS outcomes, 7 articles with inappropriate study subjects, and 5 non-RCT articles. Ultimately, this NMA included 17 studies [11, 21–23, 27–39] with a combined study population of 3470 (Fig. 1), comparing the effectiveness of 24-form, Yang style, Sun style, TCEP, and control groups on preventing falls in older adults. The characteristics of the selected articles are shown in Table 2.

**Table 2:** Basic characteristics of the included studies

Study	Sample size, n	Age, year (mean ± SD)		Tai Chi intervention	Control group	Treatment course	Primary outcomes
	Tai Chi/Control	Tai Chi	Control				
Hong et al., 2018	37/35	71.26 ± 7.95	69.97 ± 8.03	24-form simplified Tai Chi	Wellness Education	6 months	①
Li et al., 2005	125/131	76.94 ± 4.69	77.99 ± 5.14	24-form simplified Tai Chi	Stretching	6 months	①②
Li et al., 2018	224/223	77.5 ± 5.6	77.8 ± 5.9	Yang style Tai Chi	Stretching	24 weeks	①
Hwang et al., 2016	182/175	72.0 ± 8.1	72.7 ± 8.1	Yang style Tai Chi	Lower extremity training	6 months	①
Sattin et al., 2005	108/109	80.4 ± 3.1	80.5 ± 3.2	Yang style Tai Chi	Wellness Education	48 weeks	①
Taylor et al., 2012	233;220/231	75.3 ± 7.0; 74.4 ± 6.2	73.7 ± 6.2	Sun style Tai Chi	Low-level exercise	20 weeks	①
Day et al., 2015	204/205	77.6 ± 5.1	77.8 ± 5.0	Sun style Tai Chi	Flexibility and stretching program	24 weeks	①
Choi et al., 2005	29/30	76.96 ± 7.7	78.73 ± 6.9	Sun style Tai Chi	NA	12 weeks	①
Faber et al., 2006	78/90	84.4 ± 6.4	84.9 ± 5.9	TCEP	NA	20 weeks	①
Zong 2022	11/11	68.58 ± 4.55	69.34 ± 3.12	24-form simplified Tai Chi	NA	24 weeks	②
Hu 2021	15/16	66.4 ± 1.7	66.7 ± 1.8	Yang style Tai Chi	Wellness Education	16 weeks	②
Manor et al., 2014	29/28	87 ± 5	86 ± 6	Yang style Tai Chi	Education	12 weeks	②
Logghe et al., 2009	138/131	77.5 ± 4.7	76.8 ± 4.6	Yang style Tai Chi	Usual care	13 weeks	②
Saravanakumar et al., 2014	8/10	81.1 ± 8.0	85.4 ± 9.1	TCEP	Usual care	14 weeks	②
Dai 2012	48/20	68.10 ± 5.27	69.30 ± 5.91	TCEP	Walking	12 weeks	②
Wolf et al., 2003	145/141	80.9 ± 6.6	80.8 ± 5.8	Yang style Tai Chi	Wellness Education	48 weeks	①
Penn et al., 2019	20;15/15	76.45 ± 8.63; 75.27 ± 5.20	73.4 ± 8.2	TCEP; 24-form simplified Tai Chi	Education	8 weeks	②

①: Incidence of falls; ②: BBS; *SD *standard deviation, *TCEP* Tai Chi exercise program, *NA* not at all

### Assessment of risk of bias and quality of studies

**Fig. 2:**
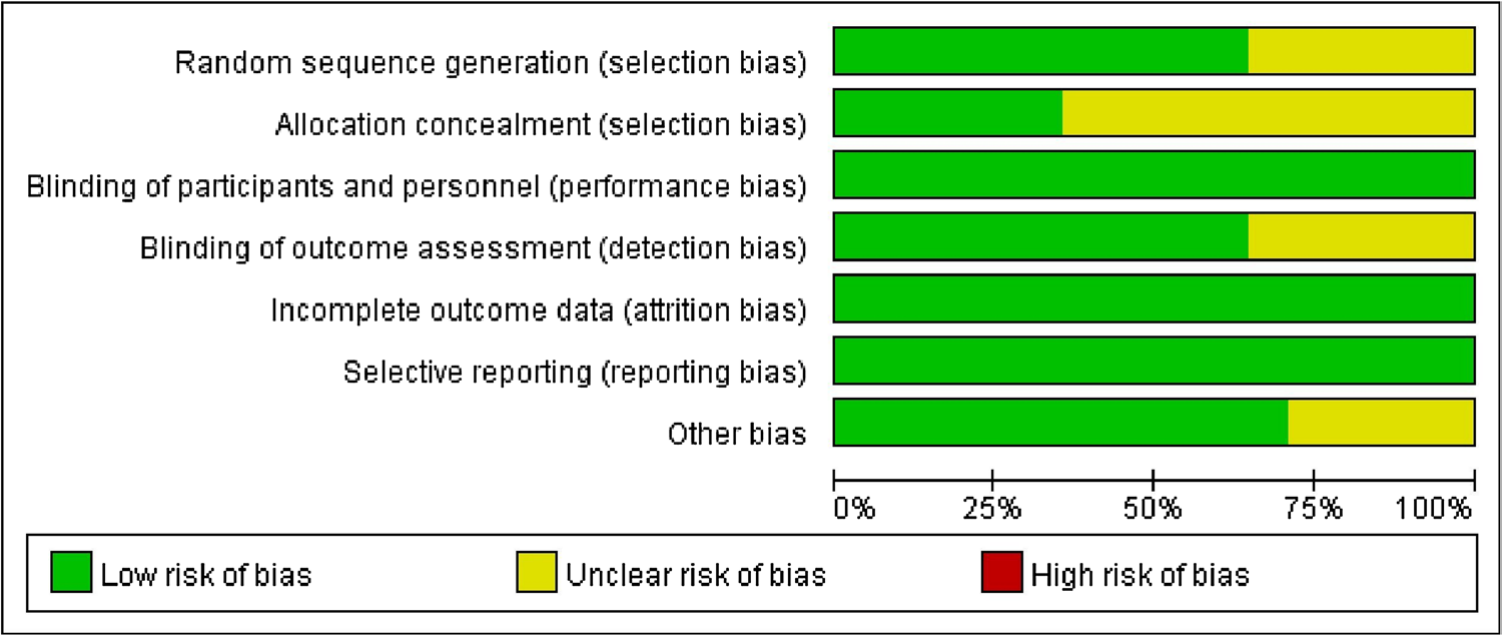
Quality assessment of included studies

The quality of selected studies was assessed according to the Cochrane Risk of Bias tool, and the results were shown in Fig. 2. 11 (65%) of the included RCTs described the process of generating random sequences using a computer or a random number table. 11 (65%) trials had an uncertain risk of allocation concealment, and all trials had a low risk of performance bias. 11 (65%) trials used a blinded approach to outcome assessment. All trials reported the expected outcome indicators (incidence of falls or BBS scores), implying a low risk of reporting bias due to selective reporting. Meanwhile, the effect values (MD) for missing data in all included trials were not sufficient to significantly affect the observed effect values, which resulted in a low risk of attrition bias for all trials.

### Results of paired meta-analysis

**Fig. 3:**
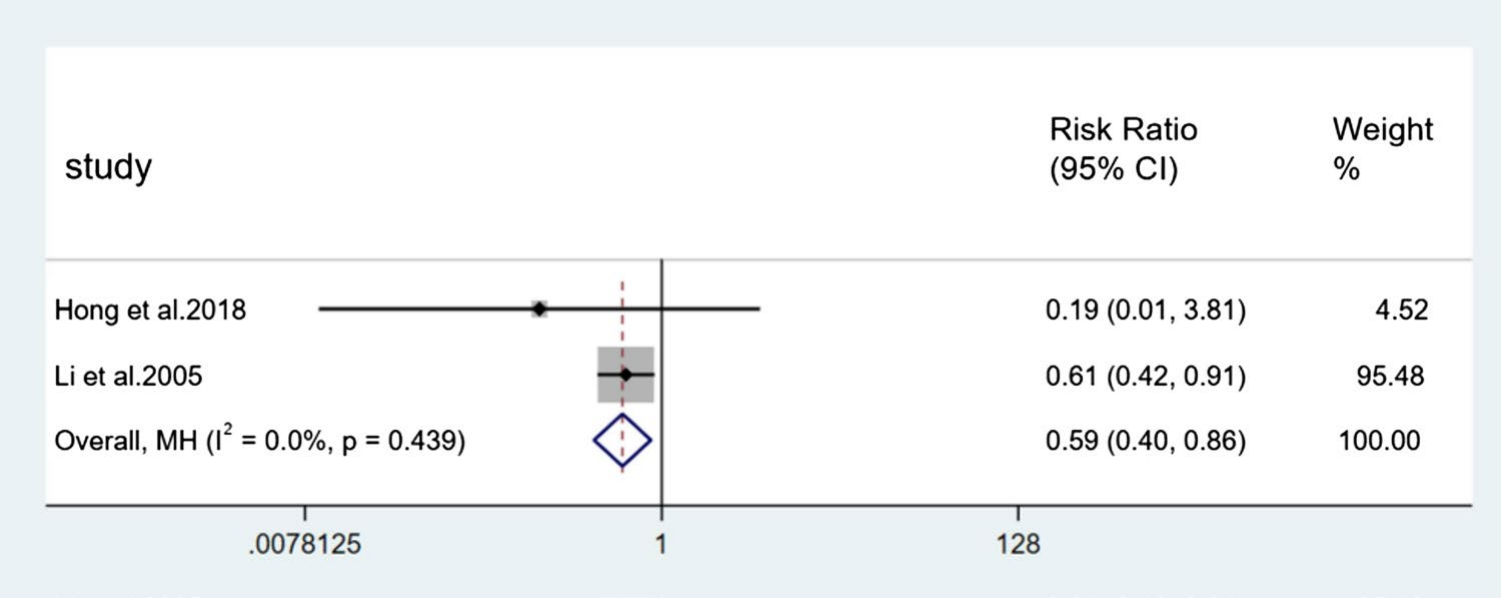
Forest plot of 24-form comparing control group in incidence of falls. *I*^*2*^% was calculated to measure the heterogeneity among studies; *CI* confidence interval

**Fig. 4:**
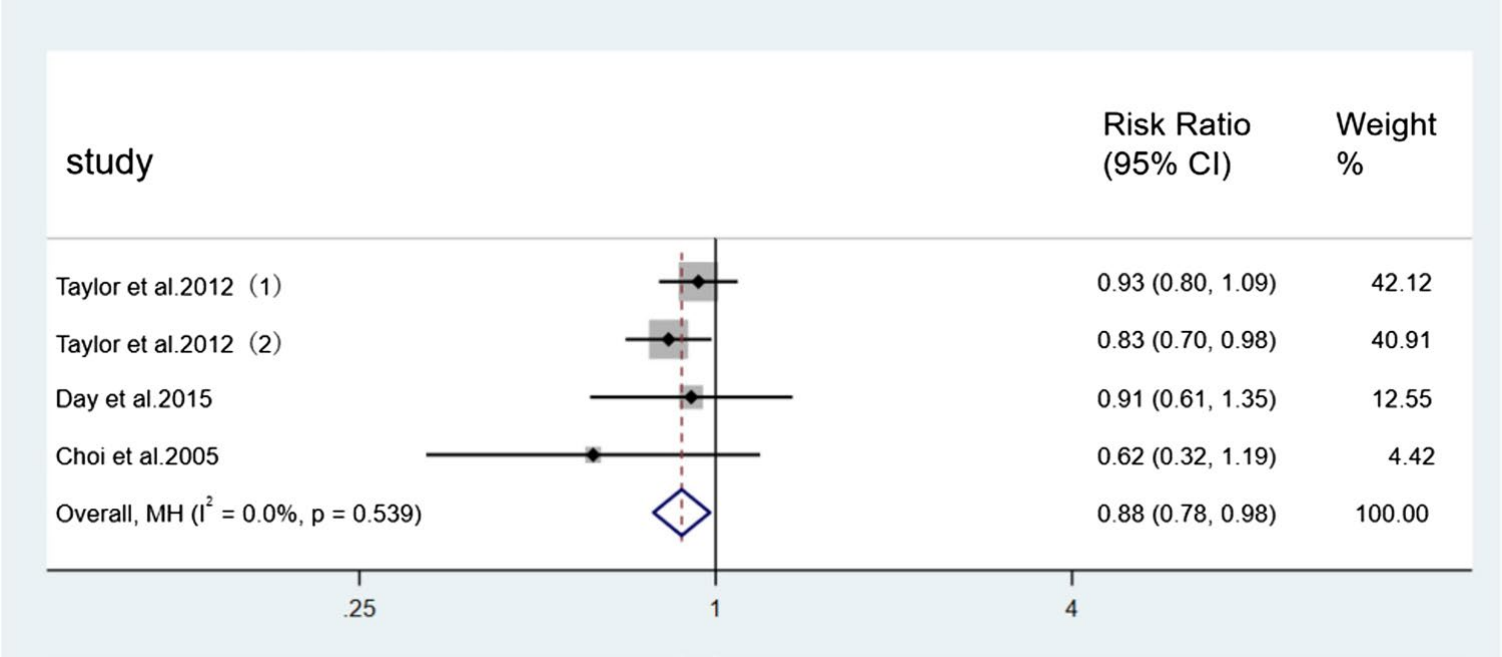
Forest plot of Sun style comparing control group in incidence of falls. *I*^*2*^% was calculated to measure the heterogeneity among studies; *CI* confidence interval

**Fig. 5:**
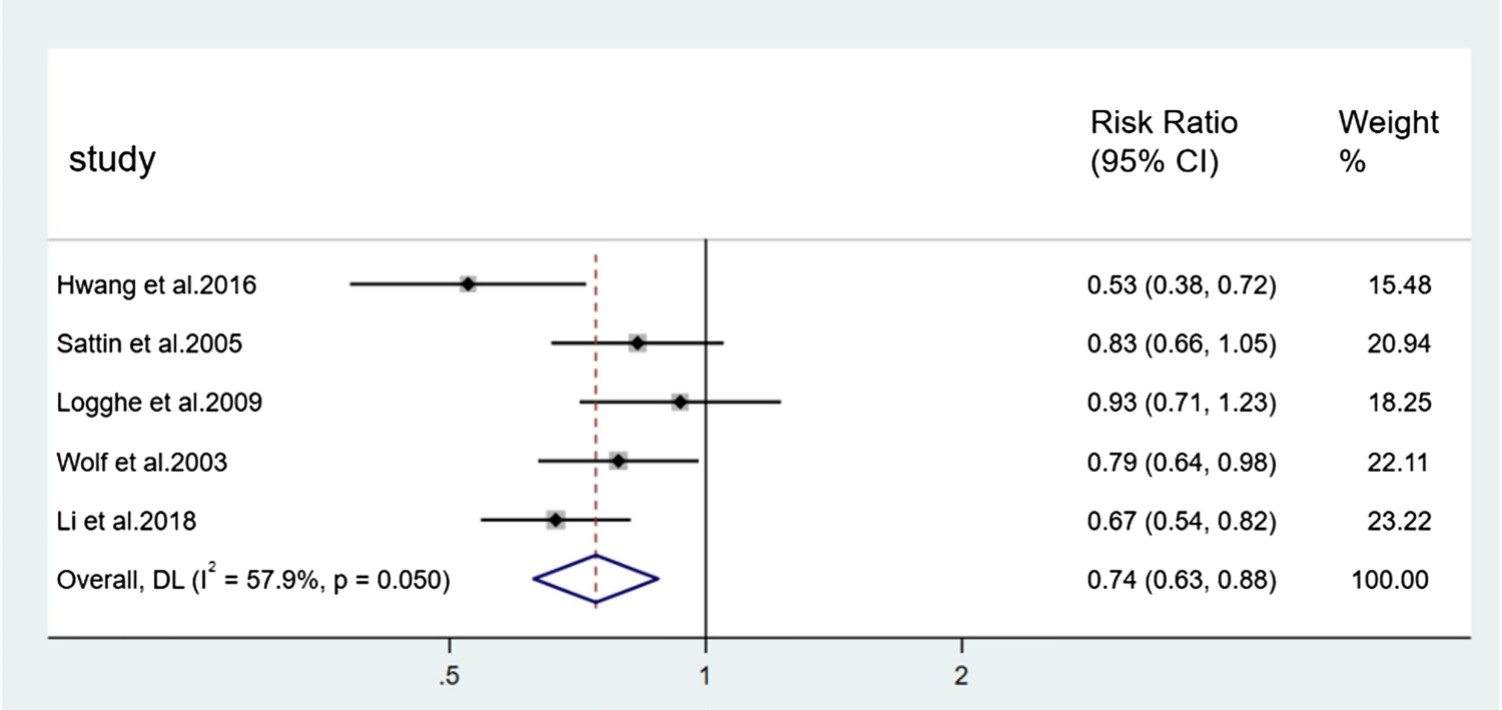
Forest plot of Yang style comparing control group in incidence of falls. *I*^*2*^% was calculated to measure the heterogeneity among studies; *CI* confidence interval

**Fig. 6:**
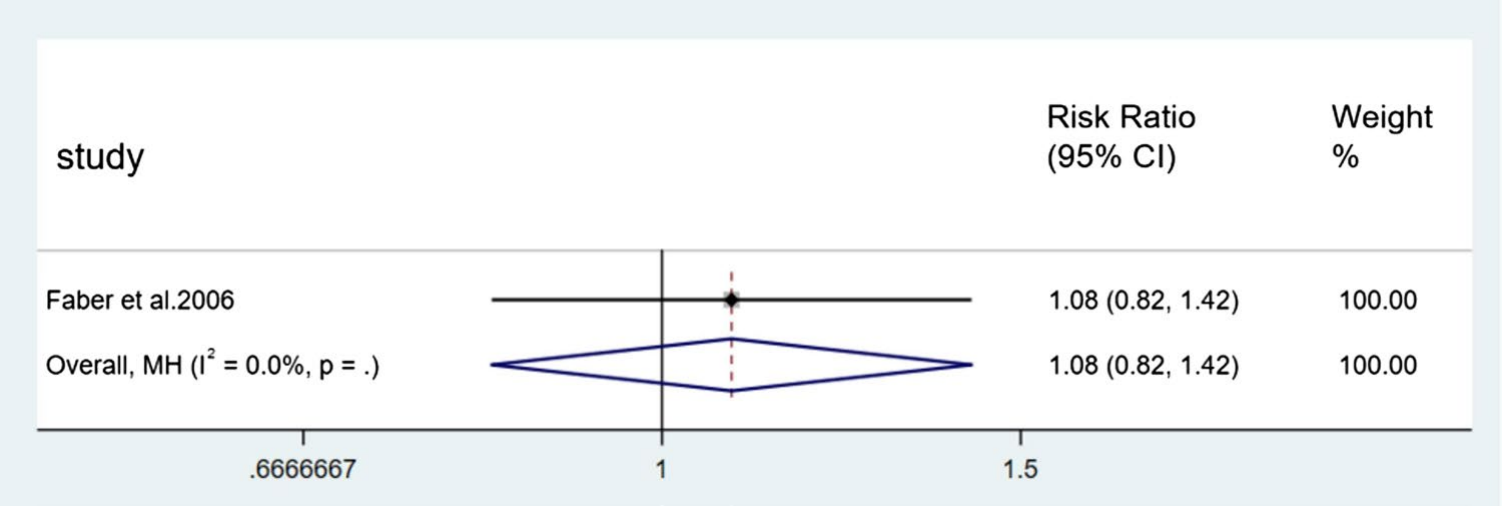
Forest plot of TCEP comparing control group in incidence of falls. *I*^*2*^% was calculated to measure the heterogeneity among studies; *CI* confidence interval

For the incidence of falls, seven direct comparisons were constructed using a fixed effect model and five direct comparisons were constructed using a random effect model. Each pair of comparisons consisted of a control group and a specific type of Tai Chi group. Results of paired meta-analysis of incidence of falls were shown in Figs. 3, 4, 5, and 6. In terms of outcome, the efficacy of 24-form (RR = 0.59, 95% CI [0.40, 0.86], *p* < 0.05, *I*^*2*^ < 50%), Sun style (RR = 0.88, 95% CI [0.78, 0.98], *p* < 0.05, *I*^*2*^ < 50%), and Yang style (RR = 0.74, 95% CI [0.63, 0.88], *p* < 0.05, *I*^*2*^ > 50%) were better than the control group. TCEP (RR = 1.08, 95% CI [0.82,1.42], *p* > 0.05, *I*^*2*^ < 50%) did not show a significant difference between TCEP and the control group.

**Fig. 7:**
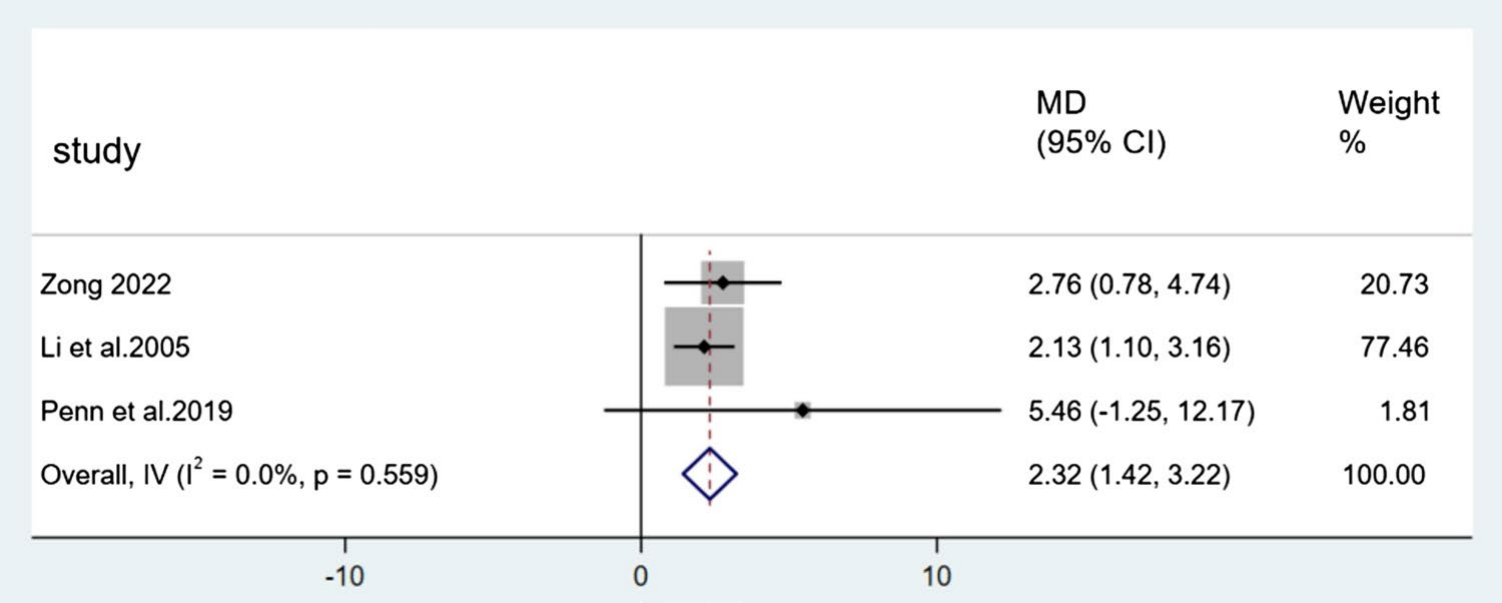
Forest plot of 24-form comparing control group in BBS. *I*^*2*^% was calculated to measure the heterogeneity among studies. *MD* mean difference, *CI* confidence interval

**Fig. 8:**
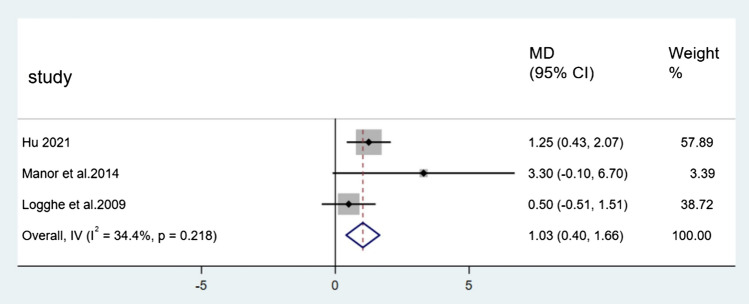
Forest plot of Yang style comparing control group in BBS. *I*^*2*^% was calculated to measure the heterogeneity among studies. *MD* mean difference, *CI* confidence interval

**Fig. 9:**
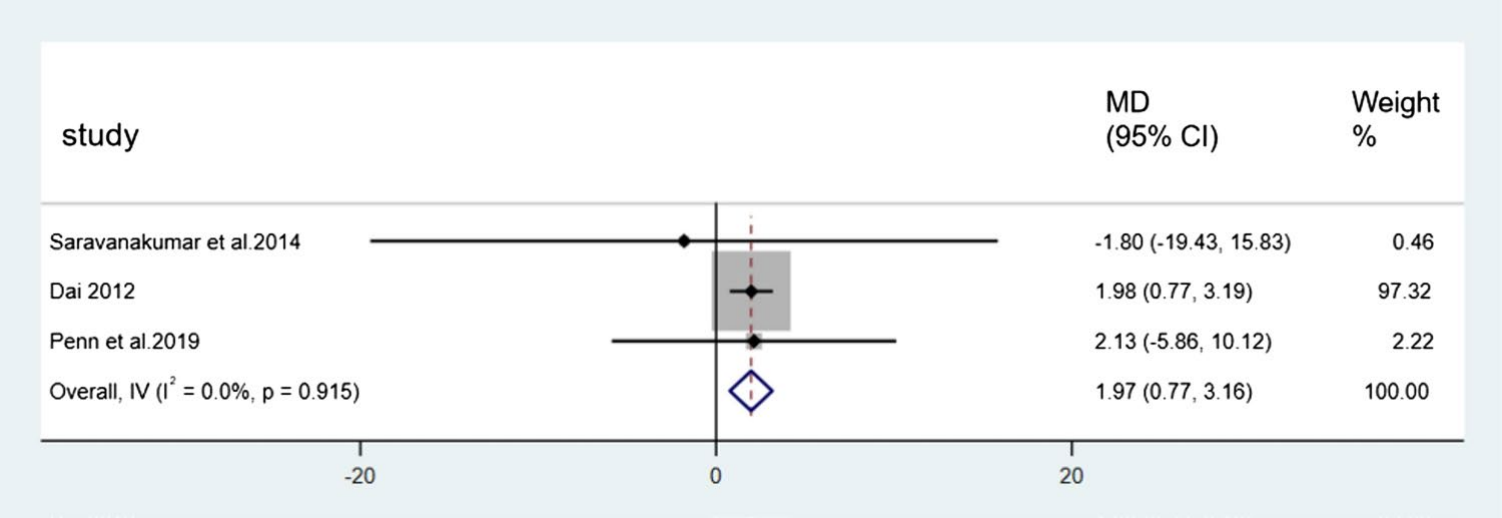
Forest plot of TCEP comparing control group in BBS. *I*^*2*^% was calculated to measure the heterogeneity among studies. *MD* mean difference, *CI* confidence interval

For BBS, Figs. 7, 8, and 9 showed the results. Nine direct comparisons were constructed using a fixed effect model. The 24-form (MD = 2.32, 95% CI [1.42, 3.22], *p* < 0.05, *I*^*2*^ < 50%), Yang style (MD = 1.03, 95% CI [0.40, 1.66], *p* < 0.05, *I*^*2*^ < 50%) and TCEP (MD = 1.97, 95% CI [0.77, 3.16], *p* < 0.05, *I*^*2*^ < 50%) were better than the control group.

### Results of NMA

#### Incidence of falls

**Fig. 10:**
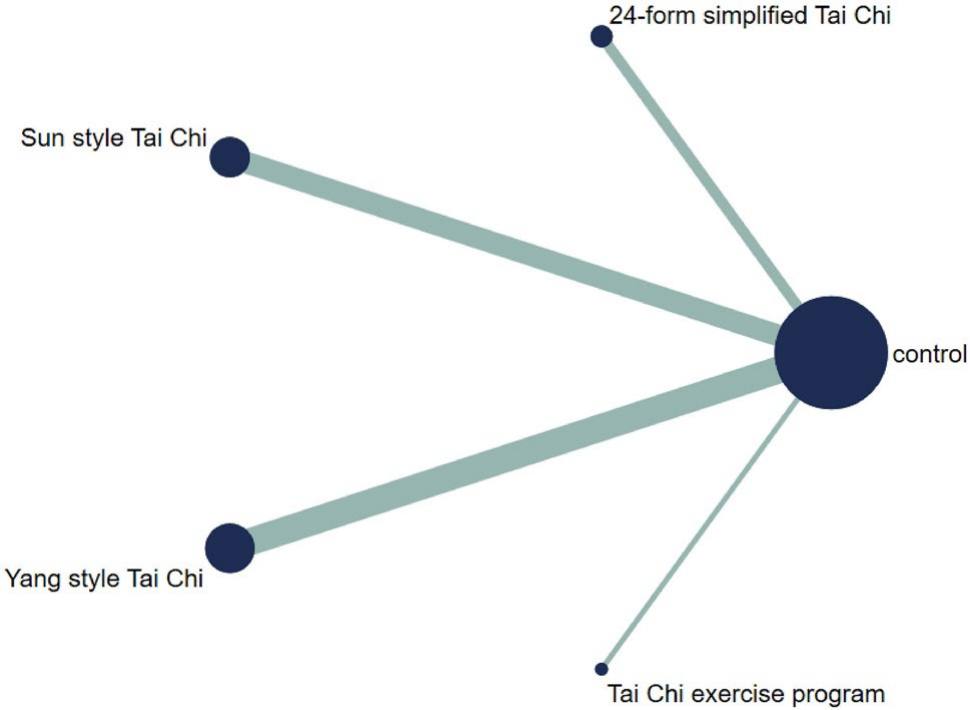
The network structure of the analyzed treatment comparisons for the outcome of incidence of falls

**Fig. 11:**
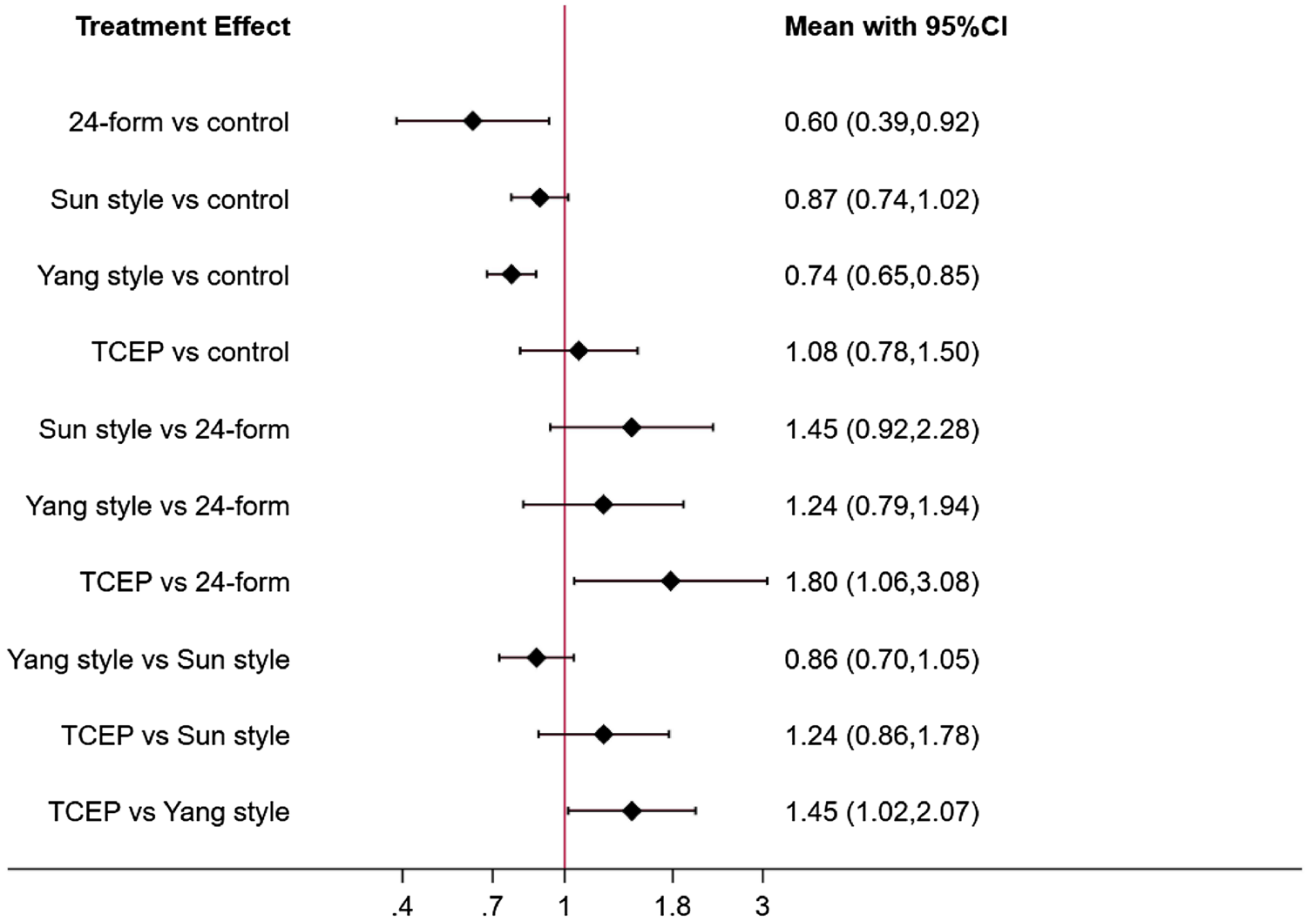
Interval plot comparing the effectiveness of the four treatments. *24-form* 24-form simplified Tai Chi, *Sun style* Sun style Tai Chi, *Yang style* Yang style Tai Chi, *TCEP* Tai Chi exercise program, *95% CI* 95% confidence interval

**Fig. 12:**
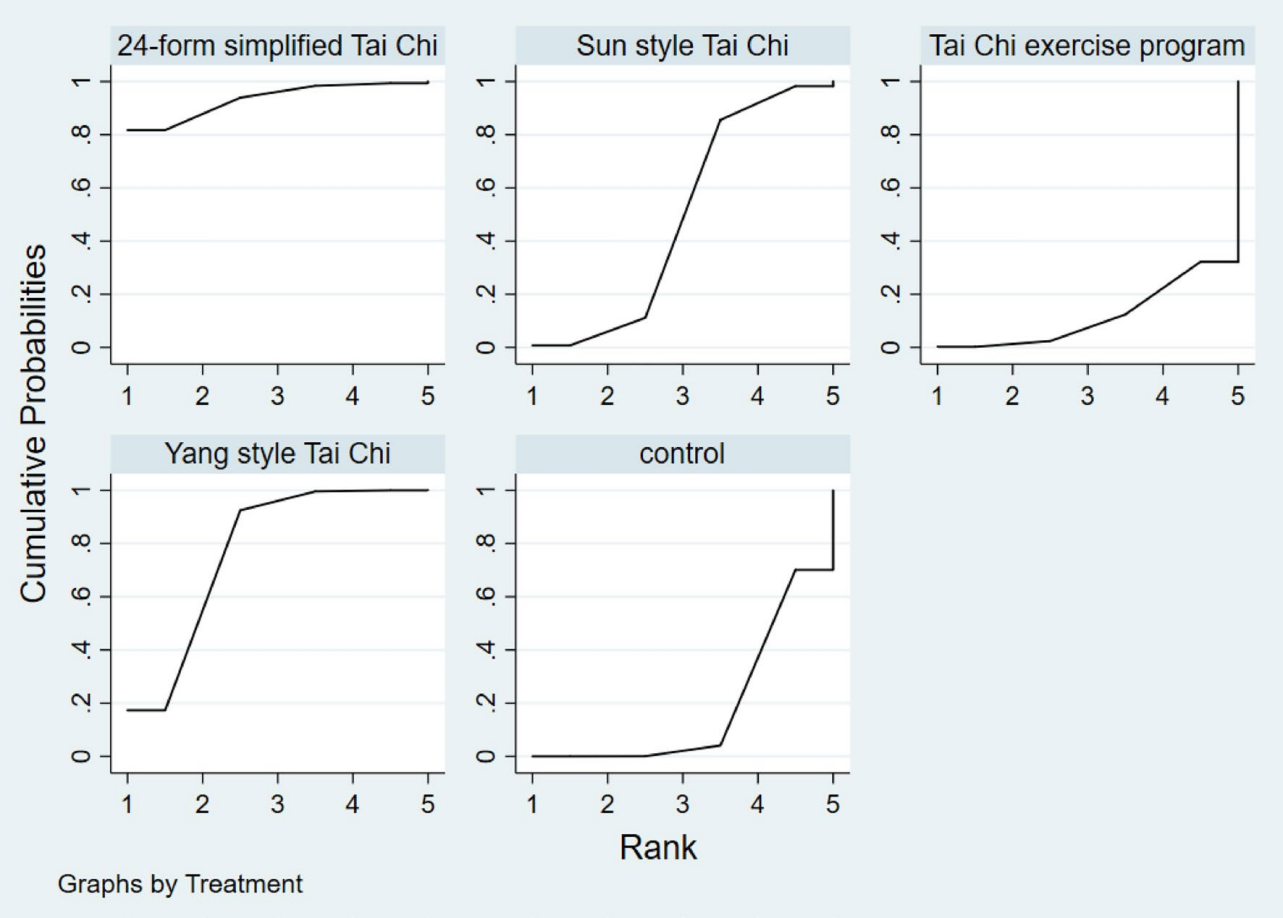
Incidence of falls rank probability

**Fig. 13:**
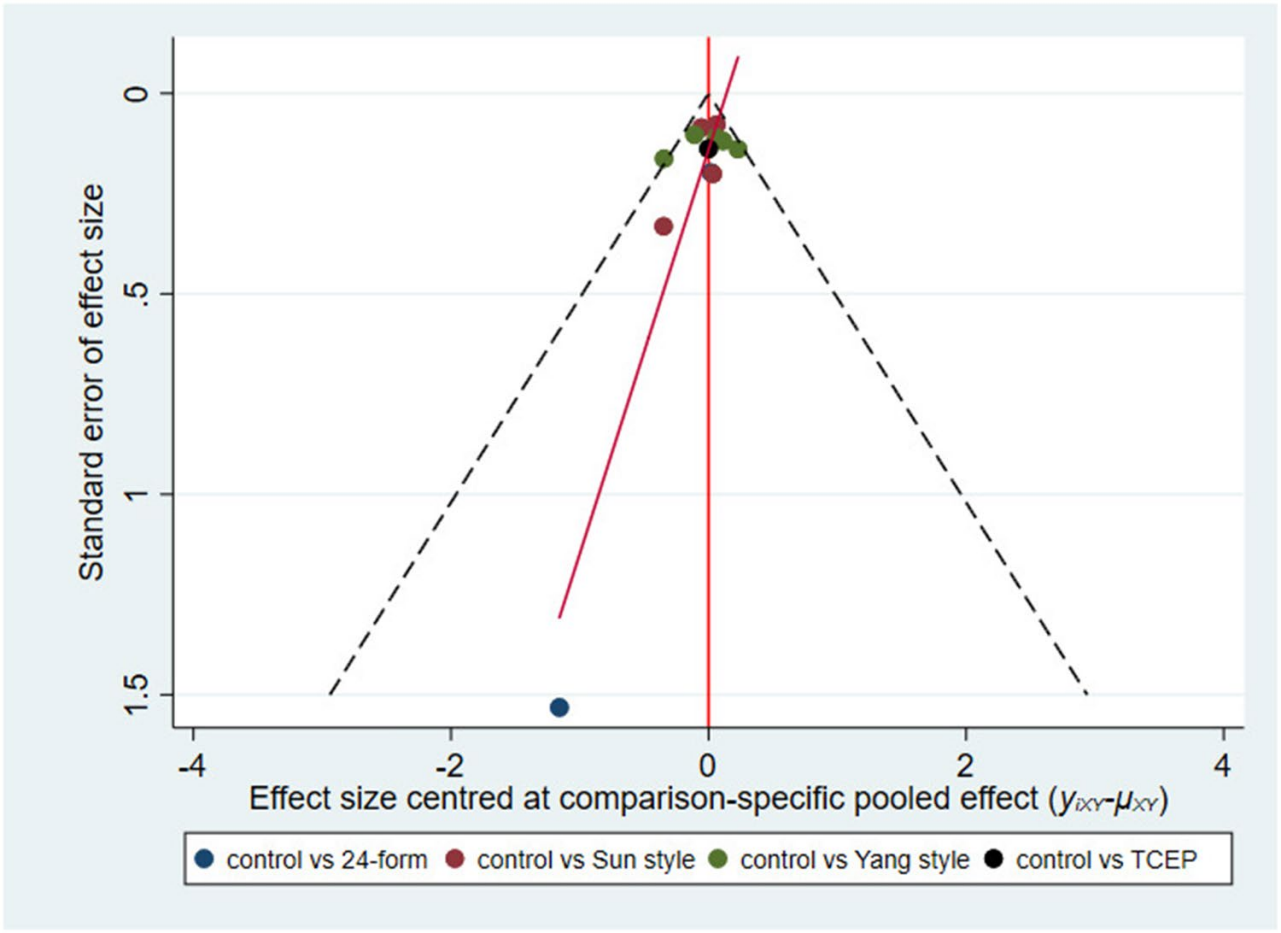
Incidence of falls: Funnel plot showing the publication bias of the included randomized controlled trials (RCTs). *24-form* 24-form simplified Tai Chi, *Sun style* Sun style Tai Chi, *Yang style* Yang style Tai Chi, *TCEP* Tai Chi exercise program

Figure 10 showed the relationship between the four types of Tai Chi in a network diagram, two RCTs comparing 24-form with the control group, four RCTs comparing Sun style with the control group, five RCTs comparing Yang style with the control group, and one RCT comparing TCEP with the control group. Since there were no loops in the network diagram, we directly used the consistency model to compare these interventions [40]. The interval plot (Fig. 11) showed that 24-form (RR = 0.60, 95% CI [0.39, 0.92]) and Yang style (RR = 0.74, 95% CI [0.65, 0.85]) were better than the control group. The rank results were as follows (Fig. 12): 24-form > Yang style > Sun style > control > TCEP. Regarding the funnel plot, the scattered distribution of the funnel plot suggested a possible small sample effect or publication bias in the study (Fig. 13). Later, Egger’s test showed no publication bias (*p* = 0.833 > 0.1).

#### Berg balance scale

**Fig. 14:**
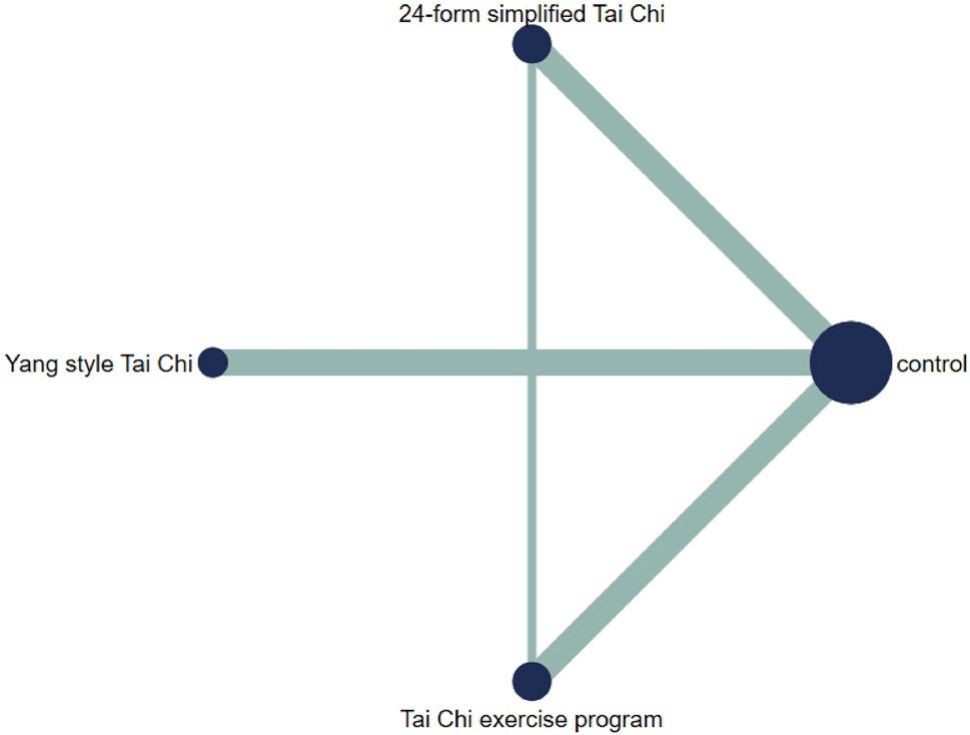
The network structure of the analyzed treatment comparisons for the outcome of BBS

**Fig. 15:**
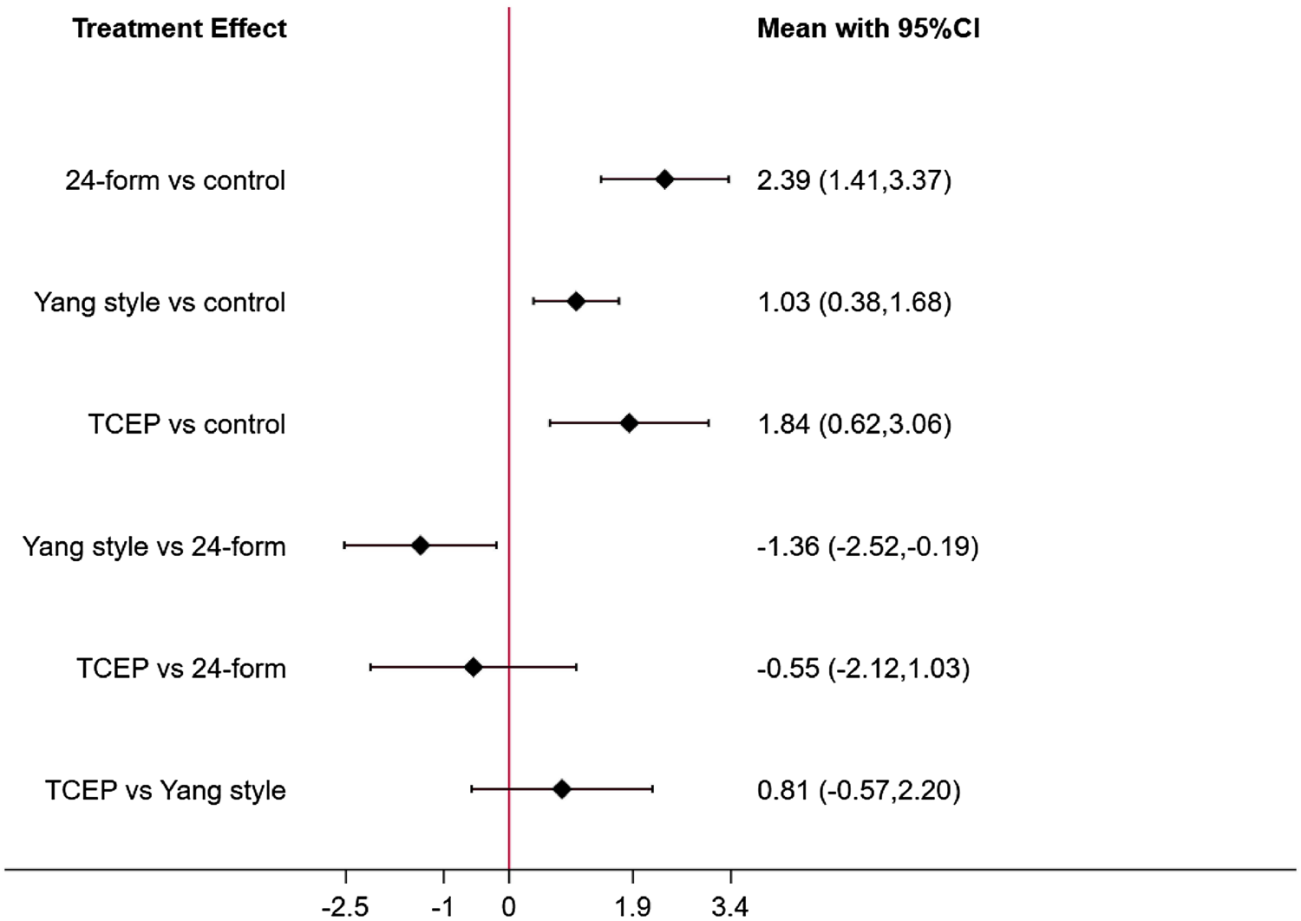
BBS: Interval plot comparing the effectiveness of the four treatments. *24-form* 24-form simplified Tai Chi, *Yang style* Yang style Tai Chi, *TCEP* Tai Chi exercise program, *95% CI* 95% confidence interval

**Fig. 16:**
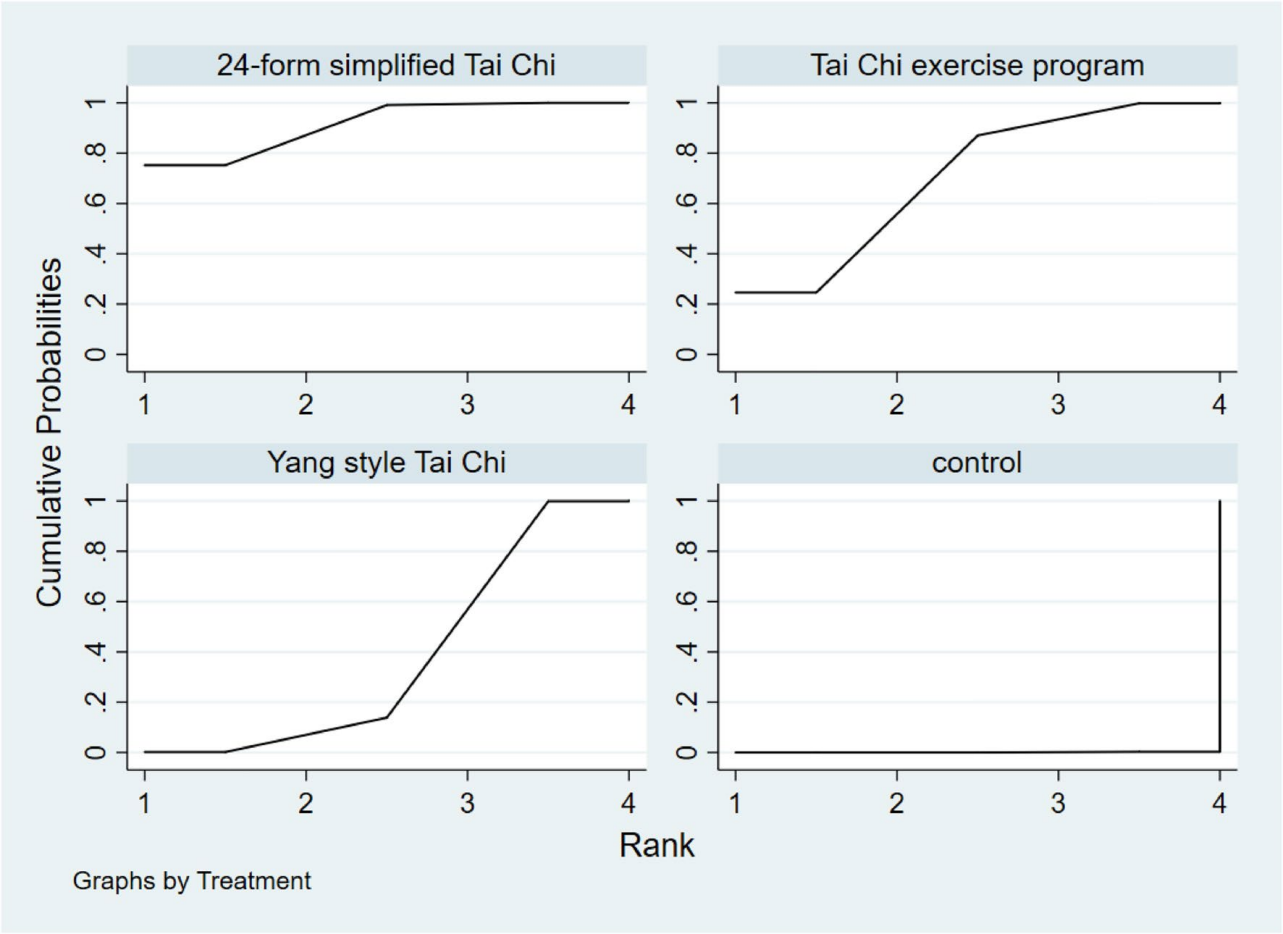
BBS rank probability

**Fig. 17:**
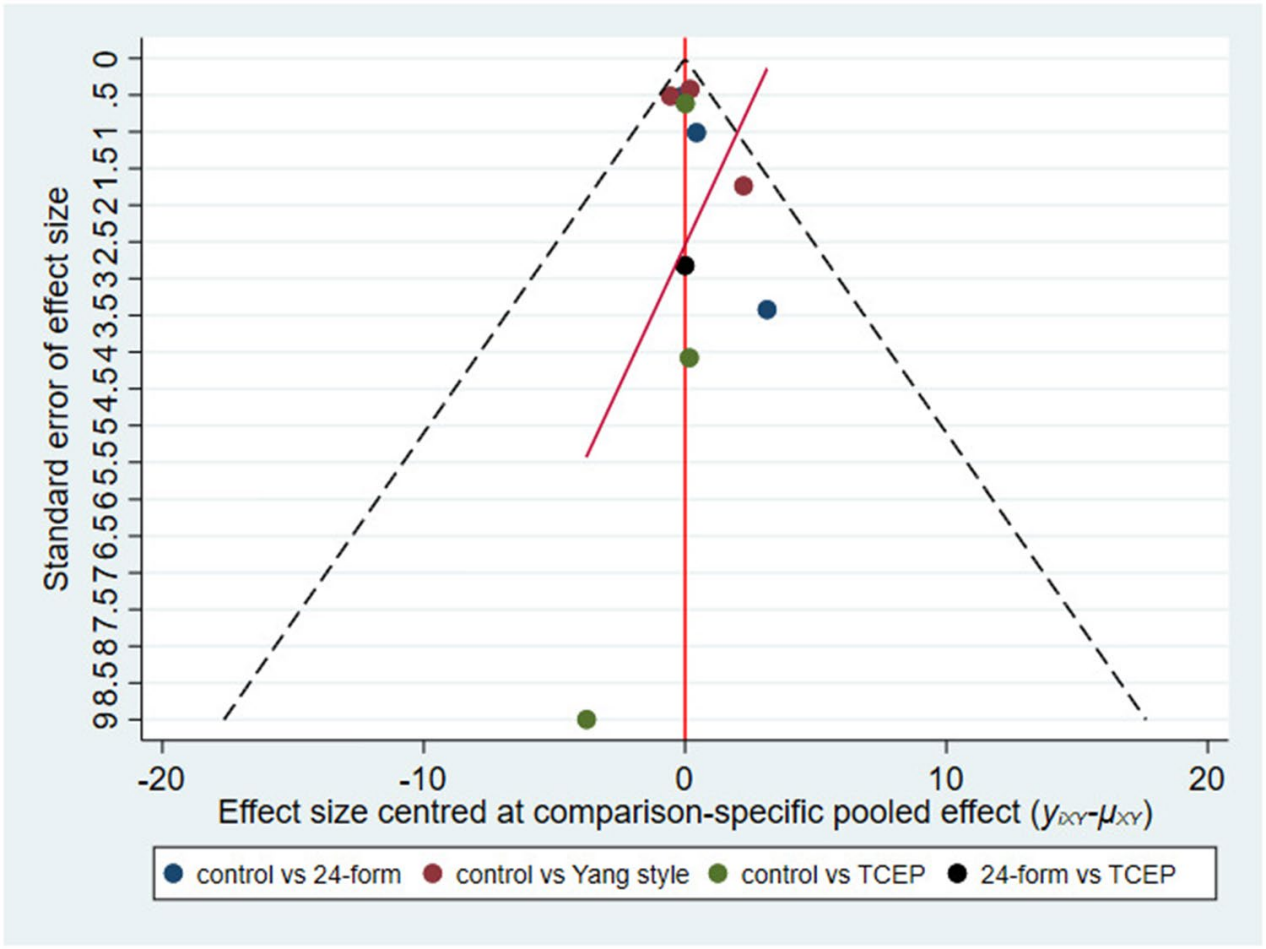
BBS: Funnel plot showing the publication bias of the included RCTs. *24-form* 24-form simplified Tai Chi, *Yang style* Yang style Tai Chi, *TCEP* Tai Chi exercise program

Figure 14 showed the relationship between the three types of Tai Chi in a network diagram, three RCTs comparing 24-form with the control group, three RCTs comparing Yang style with the control group, and three RCTs comparing TCEP with the control group. The inconsistency test was performed and *p* > 0.05, indicating that there was no inconsistency. So, we used the consistency model to analyze. The interval plot (Fig. 15) showed that 24-form (MD = 2.39, 95% CI [1.41, 3.37]), Yang style (MD = 1.03; 95% CI [0.38, 1.68]), TCEP (MD = 1.84, 95% CI [0.62, 3.06]) were better than the control group. The rank results of BBS were as follows: 24-form > TCEP > Yang style > control (Fig. 16). For the inconsistency test of the rings, *p* > 0.1, indicating that there is no inconsistency in the rings. The scatter distribution of the funnel plot indicated that the study may have a small sample effect or publication bias (Fig. 17). Later, Egger’s test indicated no publication bias (*p* = 0.402 > 0.1).

## Discussion

On the basis of 17 RCTs including 3470 participants, this systematic review indicated that 24-form Tai Chi was the most effective for preventing falls in older adults. The SUCRA values for incidence of falls were ranked from high to low: 24-form > Yang style > Sun style > control > TCEP. The BBS rank was as follows: 24-form > TCEP > Yang style > control.

An important advantage of this study is that a comprehensive literature search was conducted. On the effect of preventing falls, a previous study [41] suggested that the effect of Yang style Tai Chi was likely to be larger than the effect of Sun style Tai Chi. Our results confirm that view, which further increases our confidence in the results of the study.

For both incidence of falls and BBS outcomes, 24-form had better efficacy than the other types. Many studies [42–44] have shown that 24-form can improve balance and have a positive effect on fall prevention. Lei et al. [40] performed an NMA, and the results of BBS outcome showed that 24-form was more effective than 8-form YS, 8-form CS and control group. Our results of paired meta-analysis showed that 24-form was more effective than the control group. And the results of NMA showed that 24-form is the most effective. This is the same as those studies. The 24-form consists of slow, smooth, and rhythmic movements that put an emphasis on trunk rotation, weight shifting, coordination and a gradual narrowing of the lower extremity stance [45, 46]. The whole exercise process of 24-form is performed alternately by one leg or two legs to support one’s body weight. Therefore, the lower extremity muscle can be relaxed and tense alternately. The key to 24-form movement is to “open the hips and bend the knees”, which not only expands the support surface but also lowers the body’s center of gravity. These movements can strengthen the elderly lower limb muscle strength and muscular endurance, improving coordination and balance [47]. Coordinated slow movements reinforce the general sensory-motor process (closed-loop control). Consequently, sensory-motor systems, including vestibular and proprioceptive systems, are enhanced and reintegrated in a more effective and efficient manner. Older adults can recover more quickly and easily after a loss of balance and thereby avoid falls [48, 49].

For incidence of falls and BBS, Yang style ranked second and third. A study [50] found that the movements of “Reverse reeling forearm” and “Wave hands like clouds” could improve the balance and joint mobility of older adults; the movements of “Part the wild horse’s mane on both side”, “Sweep over knee and Step forward” and “Fair lady weaves shuttles” were practiced alternately left and right, so that enhanced the muscle strength of lower limbs. Increasing strength of the lower limb muscle groups will make body more stable. Meanwhile, Yang style has fewer forms than 24-form and is easy to learn. So older adults can practice Yang style as an entry-level Tai Chi to increase their balance.

Sun style ranked third in incidence of falls. It is to drive the whole body with feet and move the whole body with steps. The movements are centered on the lower back and hips. Its stepping style is good for developing the practitioner’s ability to change the center of gravity [20]. Moreover, Sun style has been shown to improve knee and ankle flexor and extensor muscle strength, flexibility, and mobility among older people in residential care [32].

In our study, options for the movements of TCEP were diverse, and different protocols may lead to different outcomes. One study [11] paid attention to somatosensory feedback signals coming from ankle and hip motions that can be used as input for balance control. Two studies [21, 22] focused on the modifications incorporating performance according to individual capability and safe level of comfort. One study [23] was aimed at improving balance and cardiorespiratory capacity with the selection of “Wave hands like clouds”.

However, there were some other potential limitations in our study. In terms of the incidence of falls, only the TCEP had statistically insignificant difference compared to the control group. We speculated that it may be because only one study was included in. The reason we used incidence of falls and BBS as outcome measures was because of the small amount of literature using other consistent outcome measures. Proprioception, plantar tactile sensitivity, and muscle strength are also important causes of falls. Therefore, it is suggested that clinically relevant trials could be done and perhaps more findings could be found. Considering the above limitations, our findings should be interpreted with caution.

## Conclusion

In summary, according to our study, 24-form is the most recommended for those older adults to prevent falls. We can conclude that as a moderate intensity aerobic exercise [51], 24-form is beneficial for muscle strength, balance, physical coordination, and postural stability for older adults. The United Nations (UN) General Assembly declared 2021–2030 the UN Decade of Healthy Ageing. Our findings provide new insights into exercise therapy for older adults to prevent falls. In the background of aging, there are great spaces to spread Tai Chi. It is necessary for relevant staff to provide more health education. Due to the limitation of the number and quality of included studies, the above conclusions need to be confirmed by more high-quality studies.

## Data Availability

The data used and/or analyzed during the current study is available from the corresponding author on reasonable request.
